# Role of Polymer Micelles in the Delivery of Photodynamic Therapy Agent to Liposomes and Cells

**DOI:** 10.3390/cancers12020384

**Published:** 2020-02-07

**Authors:** Laure Gibot, Maxime Demazeau, Véronique Pimienta, Anne-Françoise Mingotaud, Patricia Vicendo, Fabrice Collin, Nathalie Martins-Froment, Stéphane Dejean, Benjamin Nottelet, Clément Roux, Barbara Lonetti

**Affiliations:** 1 Laboratoire des IMRCP, Université de Toulouse, CNRS UMR 5623, Université Toulouse III—Paul Sabatier, F-31062 Toulouse, France; gibot@chimie.ups-tlse.fr (L.G.); maxime.demazeau@gmail.com (M.D.); pimienta@chimie.ups-tlse.fr (V.P.); afmingo@chimie.ups-tlse.fr (A.-F.M.); vicendo@chimie.ups-tlse.fr (P.V.); fabrice.collin@univ-tlse3.fr (F.C.); 2 Service Commun de Spectrométrie de Masse (FR2599), Université de Toulouse III (Paul Sabatier), 118, route de Narbonne, F-31062 Toulouse Cedex 9, France; martins@chimie.ups-tlse.fr; 3 IBMM, Université de Montpellier, CNRS, ENSCM, 34 090 Montpellier, France; stephane.dejean@umontpellier.fr (S.D.); benjamin.nottelet@umontpellier.fr (B.N.)

**Keywords:** Photodynamic therapy, Self-assembly, Polymer, PEO-PCL, PEO-PS, model membranes

## Abstract

The use of nanocarriers for hydrophobic photosensitizers, in the context of photodynamic therapy (PDT) to improve pharmacokinetics and bio-distribution, is well-established. However, the mechanisms at play in the internalization of nanocarriers are not well-elucidated, despite its importance in nanocarrier design. In this study, we focus on the mechanisms involved in copolymer poly(ethylene oxide)-*block*-poly(ε-caprolactone) PEO-PCL and poly(ethylene oxide)-*block*-poly styrene PEO-PS micelles - membrane interactions through complementary physico-chemical studies on biomimetic membranes, and biological experiments on two-dimensional (2D) and three-dimensional (3D) cell cultures. Förster Resonance Energy Transfer measurements on fluorescently-labelled lipid vesicles, and flow cytometry on two cancerous cell lines enabled the evaluation in the uptake of a photosensitizer, Pheophorbide *a* (Pheo), and copolymer chains towards model membranes, and cells, respectively. The effects of calibrated light illumination for PDT treatment on lipid vesicle membranes, i.e., leakage and formation of oxidized lipids, and cell viability, were assessed. No significant differences were observed between the ability of PEO-PCL and PEO-PS micelles in delivering Pheo to model membranes, but Pheo was found in higher concentrations in cells in the case of PEO-PCL. These higher Pheo concentrations did not correspond to better performances in PDT treatment. We demonstrated that there are subtle differences in PEO-PCL and PEO-PS micelles for the delivery of Pheo.

## 1. Introduction

The pharmacokinetics of a drug are typically described as four steps: Absorption, distribution, metabolism and excretion [1]. Absorption is linked to the transfer of the drug-to-blood circulation, distribution to its diffusion in the different organs and tissues, metabolism refers to its degradation through chemical reaction and excretion through physical pathways. In the last 30 years, nanomedicine has strongly developed as a possible solution for improving the therapeutic index of already-known drugs [2,3]. Indeed, numerous drugs are known to be active for certain pathologies, but are restricted by access to the site that would be treated in the most specific manner possible. Drugs might be degraded and eliminated before reaching the site, or they might diffuse in different organs and have severe side effects, as in the case of anti-cancer drugs. The encapsulation of drugs inside a nanovector is intended to overcome unfavorable pharmacokinetic properties, thereby, improving the selectivity of delivery, or increasing the efficacy by simply increasing its apparent aqueous solubility. A key element, which has prompted thousands of studies, is the discovery by Maeda in the eighties of the so-called Enhanced Permeability and Retention effect (EPR) [4,5]. This terminology describes the ability of nanovectors to spontaneously accumulate in tumor areas, due to the existence of disjunctions between the endothelial cells of blood vessels close to tumors and the strongly limited lymphatic system of tumors, preventing the rapid clearance of the vectors from the area. Compared to the tremendous hope carried by nanomedicine, only a few drug formulations are today on the market, less than 15 in the field of oncology [6]. The most successful drugs are Doxil^®^, a liposome-encapsulated doxorubicin, and Abraxane^®^, an association of albumin and paclitaxel. One of the possible explanations of this low success rate is the over-simplification of the EPR effect, which is increasingly questioned regarding its frequency [7,8,9]. An important discovery was recently made by Matsumoto et al. who observed pulses in drug transfer, which showed that the EPR might not be constant in time [10], rendering the behavior even more complex. Another recurring hypothesis is the poor understanding of the transfer of the drug from the vector to the target site. For this, the interaction between the nanovectors and the different biological barriers encountered should be better assessed [11,12]. This statement is true for all nanomedicine systems, and in particular for those developed for photodynamic therapy (PDT). PDT is based on a photosensitizer molecule, the irradiation of which locally produces reactive oxygen species, such as singlet oxygen, leading to cell death. PDT is clinically used in dermatology, ophthalmology or oncology [13,14,15]. However, limitations include the low water solubility of the photosensitizers and their tendency to form aggregates by π-π stacking, strongly decreasing their PDT efficiency. In order to improve this, encapsulation of photosensitizers has been assessed, either in liposomes [16] or in polymeric nanovectors [15,17,18,19,20,21,22]. These studies showed an improvement of the photocytotoxicity upon encapsulation, linked to a limitation of the aggregation and altered absorption by cells compared with free photosensitizer. In this context, we present here a focus on the delivery process of Pheophorbide *a* (Pheo), a known photosensitizer, from polymeric micelles based on poly (ethylene oxide-b-ε-caprolactone) (PEO-PCL) and poly (ethylene oxide-b-styrene) (PEO-PS) towards human tumor cells or model lipid vesicles. We used block-copolymer self-assemblies in previous studies [18,20,21,22] and they showed to efficiently encapsulate Pheo. Among the assessed nanocarriers, we selected micelles with approximately the same size, but with different nature of the hydrophobic block in order to discriminate the effects of these parameters in the nanocarrier-membranes interactions. Our study brings translational information between a classical biological and physical chemistry approaches.

## 2. Results

The copolymer micelles, used in this study, were formed by poly (ethylene oxide-b-ε-caprolactone) (PEO-PCL) and poly (ethylene oxide-b-styrene) (PEO-PS), using a well-established protocol [18,20,21,22], where the copolymer and the Pheo in acetone were added drop by drop to phosphate buffer solution. The micelles size, 29 ± 2 nm and 21 ± 2 nm for PEO-PCL and PEO-PS respectively, has been characterized by Dynamic Light Scattering. The Pheo and the copolymer were added in a 1/30 mole ratio in order to have the maximum content of Pheo solubilized in the hydrophobic core in its monomer form as proved by UV/VIS absorption spectra [23,24] and fluorescence lifetime measurements (Figures S2–S6 and Table S1).

### 2.1. Pheophorbide a Delivery from the Nanocarriers to Biomimetic Membranes and Human Cells

**Figure 1:**
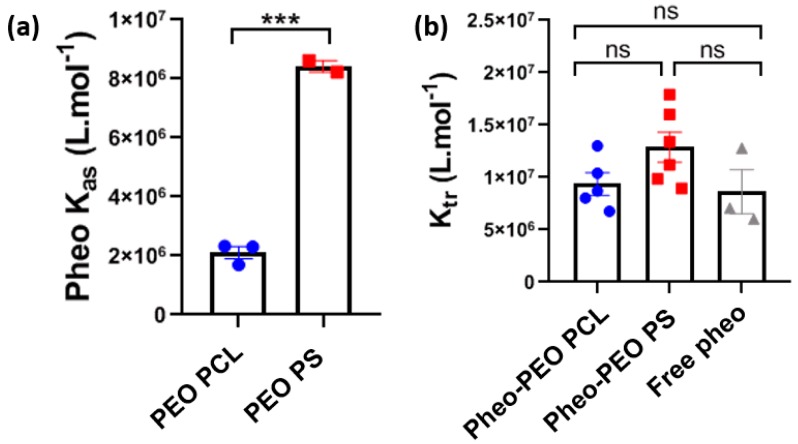
The transfer of Pheophorbide from PEO-PCL and PEO-PS micelles to Large Unilamellar Vesicles LUVs. (**a**) Determination of Pheo association constant, $K_{as}$, for the polymer micelles assessed by fluorescence experiments. Statistical analysis by t-test, *** = *p* < 0.001 (**b**). The transfer of Pheo from PBS solution (free Pheo) and Pheo loaded copolymer micelles to liposomes assessed by Förster Resonance Energy Transfer. $K_{tr}$ is the constant of Pheo transfer in the different conditions. Statistical analysis by One-way Anova followed by Tukey’s multiple comparisons test. ns = non-significant; Data are represented as the mean value ± SEM.

Figure 1a represents the association constant of the photosensitizer Pheo towards the two used block-copolymers, PEO-PCL and PEO-PS, organized into micelles. In a typical experiment, different quantities of the copolymers (concentration between 10^−7^ and 10^−4^ M) were added to a Pheo solution at 10^−8^ M in PBS and the fluorescence intensity was measured at 675 nm. A representative set of fluorescence spectra is reported in Figure S1. When Pheo was in its monomer form, free in aqueous solution, the fluorescence signal was as expectedly low [25] and then progressively increased upon micelle addition, as Pheo progressively entered the hydrophobic environment of the nano-objects. Once Pheo is encapsulated in the nano-objects, it is in monomer form, as witnessed by the absorbance spectrum in the presence of the amphiphilic molecules, which is very similar to those obtained in an organic solvent, such as ethanol and by the fluorescence lifetime values (see Figures S2–S6 and Table S1). According to Kuzelova et al. [26] an equilibrium can be written between Pheo free in solution and the empty copolymers organized in nano-objects and that encapsulated in the nano-objects as follows,

(1) pheo+Pol⇆pheo:Pol

where *pheo* stands for Pheo in the bulk solution, *Pol* is the copolymer and *pheo:Pol* is Pheo associated to the amphiphilic species. The fluorescence intensity at a given wavelength (λ = 675 nm) can be reported as a function of the copolymer concentration, in order to determine the equilibrium association constant (K_as_):

(2) $$K_{as}=\frac{\left[pheo:Pol\right]}{\left[pheo\right]\times \left[Pol\right]}.$$

The copolymer concentration is known and Pheo is both free in solution and associated to the micelles:

(3) $${\left[pheo\right]}_{TOT}=\left[pheo\right]+\left[pheo:Pol\right].$$

Since Pheo is the only fluorescent species, the total fluorescence signal is the sum of the contributions of bulk Pheo and that inside the nano-objects. At the concentration used, Pheo was in monomer form [27] and the fluorescence signal is directly proportional to its concentration for both species,

(4) $$F_{pheotot}=F_{pheo}+F_{pheo:Pol}=\;f_{pheo}\left[pheo\right]+f_{pheo:Pol}\left[pheo:Pol\right]$$

where $f_{pheo}$ and $f_{pheo:Pol}$ are the fluorescence proportionality constants linking the fluorescence intensity of the Pheo species to their concentrations. $f_{pheo}$= 2.1 × 10^12^ M^−1^ has been experimentally evaluated from the spectra before the addition of the nano-objects and fixed in the fitting procedure. $f_{pheo:Pol}$ and $K_{as}$ are the unknown parameters adjusted through the fitting procedure.

The obtained association constants are shown in Figure 1a and indicate that Pheo is located preferably in the PS hydrophobic core with respect to PCL. This is reasonable as the aromatic units of the PS chains could present π stacking interactions with the porphyrin group of Pheo. This result is consistent with already published results comparing the Pheo release from PEO-PCL and PEO-PS polymeric micelles in dialysis experiments [18].

The transfer of the photosensitizer from the nanocarrier to the membranes is related to its affinity for the copolymers, but also to the way the transfer takes place. In order to estimate the tendency of the nanocarrier to deliver Pheo to the membrane, we performed Förster Resonance Energy Transfer (FRET) measurements with dioleylphosphatidylcholine (DOPC) Large Unilamellar vesicles (LUV) taken as membrane models (Figure 1b). For this purpose, rhodamine was chosen as a dye to stain the liposomes as its emission spectrum partially overlaps to the absorption spectrum of Pheo (see Figure S7a). This means that the two fluorophores form a FRET couple where the rhodamine is the donor and Pheo the acceptor. Their critical distance, R_0_ (i.e., the distance at which the energy transfer and spontaneous decay of the excited donor are equally probable) is estimated as 4 nm (see SI.II and Table S2). This means that rhodamine and Pheo must be separated by a distance equal or lower than 4 nm for the energy transfer to occur. The fluorescence emission spectra of solutions containing both rhodamine-labelled liposomes at fixed concentration (0.12 μM) and Pheo loaded micelles present a peak relative to Pheo fluorescence only when the two fluorophores are spatially close (see Figure S7b). In order to quantify and compare the two nano-objects, PEO-PCL and PEO-PS micelles respectively, the transfer process can be represented in a simplified way as,

(5) pheo+rhod⇆pheo:rhod

where *pheo* and *rhod* are the Pheo and the rhodamine at distances larger than 4 nm, and *pheo:rhod* is the Pheo/rhodamine couple undergoing resonance energy transfer. The values we use for *pheo* in this model are either the concentration of free Pheo, or the concentration of Pheo trapped in PEO-PCL or PEO-PS micelles. The associated constant (K_tr_),

(6) $$K_{tr}=\frac{\left[pheo:rhod\right]}{\left[pheo\right]\times \left[rhod\right]}$$

can be obtained in a similar way as for the association constant K_as_, from the plot of rhodamine maximum fluorescence intensity (λ = 591 nm) decrease as a function of the copolymer concentration (see Figure S8). In the investigated concentration range, the fluorescence emission (F) is proportional to the concentration of the fluorophores, thus,

(7) $$F=F_{rhod}+F_{pheo:rhod}=\;f_{rhod}\left[rhod\right]+f_{pheo:rhod}\left[pheo:rhod\right]$$

(8) $${\left[pheo\right]}_{TOT}=\left[pheo\right]+\left[pheo:rhod\right]$$

$F_{rhod}$, $F_{pheo:rhod}$ represent the fluorescence intensity of rhodamine and Pheo coupled to rhodamine at 591 nm. $f_{rhod}$ and $f_{pheo:rhod}$. represent the fluorescence proportionality constants linking *rhod* and *pheo:rhod* to their concentration. $f_{rhod}$ = 1.10 × 10^11^ M^−1^ was experimentally evaluated from the values of the spectrum in the absence of Pheo. $f_{pheo:rhod}$ and $K_{tr}$ are the fitted parameters. The obtained $f_{pheo:rhod}$ values are comparable for the three experiments (3.6 × 10^10^ ± 0.5 × 10^10^ M^−1^). It is noted that the model applied makes no assumption about the actual localization of Pheo and rhodamine, meaning that the Pheo could be transferred to the LUV membrane or the Rhodamine labelled lipid could insert in the micelles. It is also noted that both events imply that very intimate contact is involved between the nano-objects.

The values of the thus obtained K_tr_ were compared for Pheo in PBS and Pheo, loaded in the micelles in Figure 1b. No statistically significant difference between the values was observed, meaning that even if the association constant between Pheo and PEO-PS is higher than the one between Pheo and PEO-PCL, the differences in Pheo transferred to the biomimetic membrane were negligible due to the high affinity of Pheo towards the lipids constituting the vesicles [27]. 

**Figure 2:**
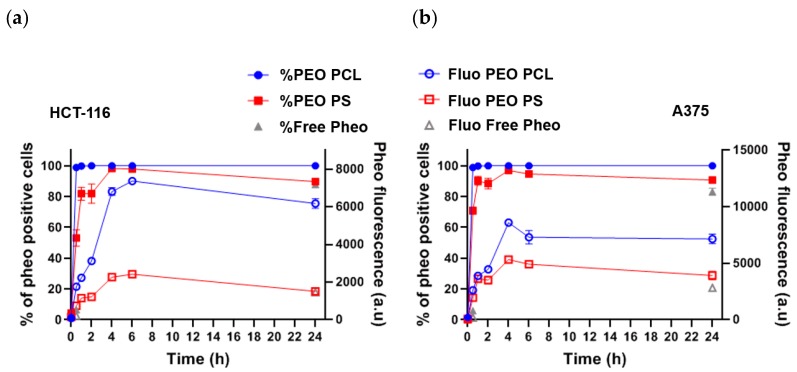
Pheophorbide fluorescence in human tumor cells exposed to Pheo loaded PEO-PCL and PEO-PS micelles. (**a**) Quantification by flow cytometry of Pheo fluorescence in HCT-116 when incubated over 24 h with Pheo in PBS (free Pheo) or Pheo loaded PEO-PCL or PEO-PS micelles. (**b**) Quantification by flow cytometry of Pheo fluorescence in A375 cells when incubated over 24 h with Pheo in PBS (free Pheo) or Pheo loaded PEO-PCL or PEO-PS micelles. *n* = 4. Data are represented as the mean value ± SEM.

Moreover, the Pheo uptake by human tumor cells was assessed by flow cytometry, which gave an indication of the total quantity of Pheo present in the cells over time (Figure 2). Two distinct cancerous cell types were used in this experiment: Human colorectal HCT-116 and human melanoma A375 cancer cell lines. Pheo penetration within cells seemed to follow the same kinetics, whichever the nanocarrier (i.e., PEO-PCL or PEO-PS micelles), with a maximum of fluorescence reached after 4 to 6 h of incubation. We observed for both cell types that 100% of the cell population were positively labelled by Pheo within the first hours of incubation with encapsulated Pheo. After 24 h of incubation with Pheo loaded micelles or free Pheo, cell fluorescence intensity was comparable between PEO-PS micelles and free Pheo, while much stronger with PEO-PCL micelles. This suggests that more Pheo was found in cells when carried by PEO-PCL micelles than with PEO-PS ones which did not differ from free Pheo. This observation seems to contradict what we observed in experiments on biomimetic membranes, but it must be underlined that acellular experiments were performed in PBS, so no other competitive absorption on hydrophobic environment could take place. This is not true in the case of cell experiments where fetal bovine serum (10%) was employed to feed the cells. We can expect that free Pheo in solution interacted with the cell culture proteins, and thus, decreases the absorption at the cell scale.

It is instructive to correlate the results on biomimetic membranes with those on human cells. Experiments on model membranes have been performed at fixed LUV concentration (the total hydrophobic volume can be estimated to 2 × 10^−3^ mm^3^) and different Pheo concentrations, in order to determine the ratio between available hydrophobic volume in liposomes and maximum Pheo concentration. From Figure S8, the saturation of the fluorescence signal indicates that the maximum number of Pheo molecules that can be accommodated in the lipid membrane is reached between 0.5 and 0.8 μM of Pheo. Experiments on human cells correspond to a fixed ratio between Pheo loaded micelles and available hydrophobic plasma membrane. In biological experiments leading to flow cytometry analyses, 80,000 cells were seeded into 12-well plates in which growth area is 3.8 cm². In order to facilitate the estimation of cell surface available to micelles, we decided not to account for cell division during the experiment. The cell mean area was measured on at least 60 independent properly adhered cells and was found to be 398 ± 100 µm² and 781 ± 180 µm² for HCT-116, and A375, cell types respectively. In this way, taking into account lipid bilayer thickness, we estimated the hydrophobic membrane volume accessible in experiments on human cells to about 0.1 × 10^−3^ mm^3^ and 0.2 × 10^−3^ mm^3^ for HCT-116 and A375 respectively, lower than the one used in experiments on lipid vesicles (2 × 10^−3^ mm^3^). In biological experiments, [Pheo] = 0.33 μM was used with a total accessible hydrophobic volume 10 to 20 times (for HCT-116 and A375 respectively) smaller than the one in the case of LUVs. This means that the experiment on human cells uses a large excess of Pheo compared with the hydrophobic volume available for its absorption. This will be discussed in greater detail in the discussion section.

### 2.2. Polymer Incorporation to Biomimetic Membranes and Human Cells

**Figure 3:**
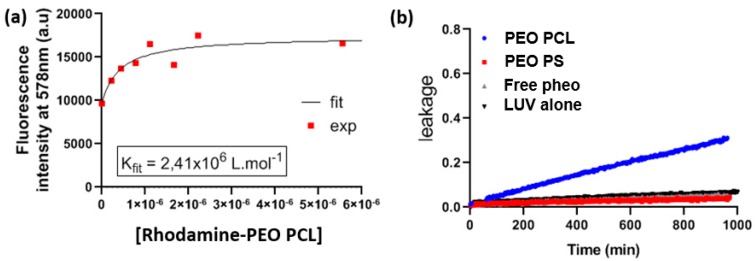
Transfer of polymers from micelles to LUVs. (**a**) Analysis of Rhodamine labelled PEO-PCL transfer from PEO-PCL micelles to NBD-LUVs, assessed by FRET. (**b**) Carboxyfluoresceine leakage from DOPC LUVs alone and DOPC LUVs challenged with free Pheo and with the PEO-PCL and PEO-PS micelles.

As the nanocarriers used are self-assembled objects, material exchange between the micelles and liposomes cannot be completely excluded. In order to get further insight on this point, we used a PEO-PCL copolymer where rhodamine was covalently linked to the PCL-end group (see Section SI.III and Figure S9 for the description of the polymer synthesis and characterization). In a similar way as for the Pheo transfer from the micelles to the LUVs, we performed FRET experiments using DOPC LUVs doped with N-(7-Nitrobenz-2-Oxa-1,3-Diazol-4-yl)-1,2-Dihexadecanoyl-sn-Glycero-3-Phosphoethanolamine (NBD-PE). NBD dye is known to form a FRET couple with rhodamine. The calculated R_0_ distance for the NBD/rhodamine couple is 4 nm, as described in Section SI.II, so the fluorescence intensity of rhodamine labeled PEOPCL increases if it is at distances lower than 4 nm from NBD dye in the membrane of LUVs. When rhodamine-labeled PEO-PCL micelles were challenged with NBD LUVs, we observed an increase of the rhodamine fluorescence already at small copolymer concentrations and the rhodamine fluorescence reaches a maximum at copolymer concentration between 3 and 6 μM (Figure 3a and Figure S10). This observation suggested a strong interaction of copolymer micelles with the biomimetic membrane (see discussion section for further details on this point). In a similar way, as for Pheo/rhodamine FRET experiments, we were able to model this increase in order to calculate a transfer constant, K_tr_ = 2.4 × 10^6^ M^−1^. This value is one order of magnitude lower than the one found for Pheo.

In order to gain insight on the effect of the copolymers themselves on the LUVs membrane integrity, we performed leakage experiments to estimate the permeability of the membranes in the different experimental conditions. For that purpose, we followed the leakage of carboxyfluoresceine (CBF) from LUVs challenged with the copolymer micelles (Figure 3b). Interestingly PEO-PCL micelles induced a marked increase of the LUVs’ membrane permeability. This is another indication of the strong interaction of PEO-PCL micelles with lipid membranes. In contrast, PEO-PS had no effect on lipid membrane permeability.

**Figure 4:**
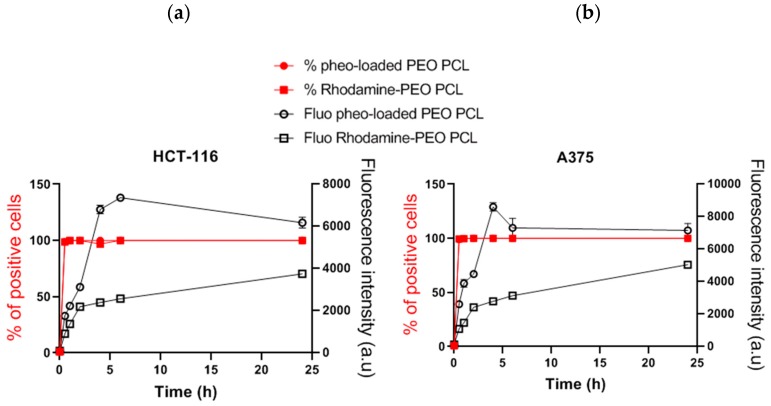
Internalization of PEO-PCL by human tumor cells. (**a**) Comparison of cell penetration kinetics of Pheo loaded PEO-PCL micelles and rhodamine labelled PEO-PCL micelles, quantified by flow cytometry in HCT-116. (**b**) Comparison of cell penetration kinetics of Pheo loaded PEO-PCL micelles and rhodamine labelled PEO-PCL micelles, quantified by flow cytometry in HCT-116. *n* = 4. Data are represented as the mean value ± SEM.

Taking advantage of polymer PEO-PCL covalently linked to rhodamine, we analyzed by flow cytometry how polymer was internalized into tumor cells after incubation with micelles over 24 h at 37 °C (square symbols in Figure 4). In both cell types, 100% of the cell population was positively labelled with rhodamine after 30 min of incubation with micelles, indicating cellular uptake of polymer. The rhodamine fluorescence of the total cell population increased over the 24 h of incubation, meaning that polymer kept penetrating into cells over time. In order to better compare the kinetics of uptake of Pheo and PEO-PCL polymer from PEO-PCL micelles to cells, an overlay of flow cytometry results is provided in Figure 4. While the percentage of cells labelled in the population was superimposed for Pheo and polymer entry, the evolution of global fluorescence differed. Pheo rapidly and massively entered the cells, reaching a maximum after 6 h before decreasing slightly. Rhodamine fluorescence increased slightly and constantly over the 24 h of the experiment. These two different kinetic regimes underlined distinct behaviors for the internalization of Pheo and polymer from micelles to cells.

### 2.3. Interactions of Pheophorbide-Loaded Micelles with LUVs under Light Irradiation

**Figure 5:**
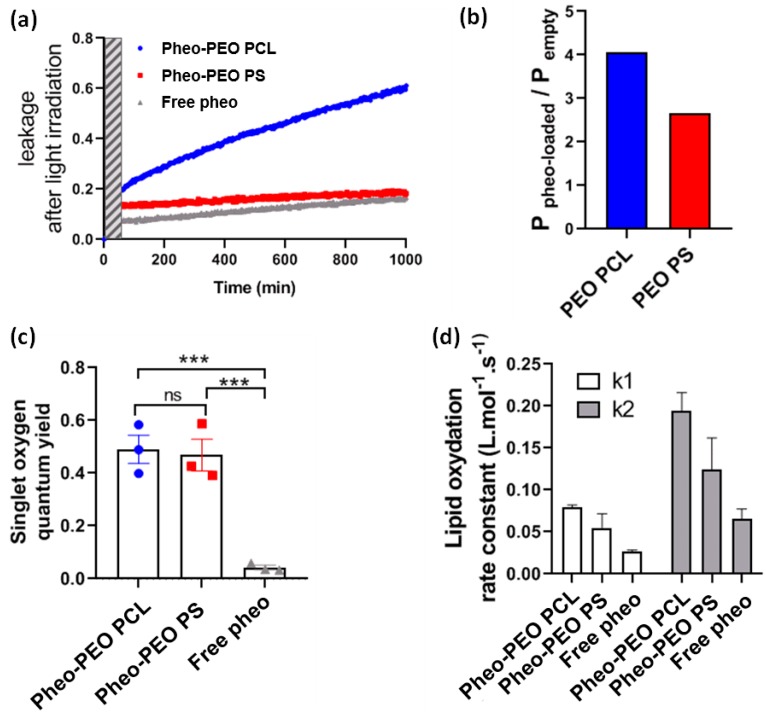
Analysis of Pheo loaded micelles interactions with liposomes under light irradiation. (**a**) The permeability of liposomes was quantified by fluorescence through carboxyfluoresceine (CBF) leakage. Dashed bar indicates light irradiation. LUV = Large Unilamellar Vesicle. (**b**) P = permeability constant. (**c**) Singlet oxygen quantum yield of free Pheo of Pheo loaded micelles quantified by spectrophotometric analysis. (**d**) Determination of oxidation rate constant from fitted UPLC-MS data. *** = *p* < 0.001.

After analysing the transfer of Pheo from the micelles to the lipid vesicles, we characterized the effect of Pheo irradiation on the integrity of biomimetic membranes, in terms of membrane permeability to small molecules. We followed the leakage of CBF from LUVs, challenged with free Pheo and Pheo-loaded micelles, after light irradiation with a LED lamp at 656 nm. Figure 5a shows the CBF-leakage from LUV challenged with Pheo in PBS and encapsulated in PEO-PCL and PEO-PS micelles. After 30 min of irradiation, 20% (Pheo loaded PEO-PCL), 14% (Pheo loaded PEO-PS) and 7% (free Pheo) of total CBF had been released from the vesicles, indicating a strong leakage during irradiation. The leakage rate diminished when irradiation was stopped. In the case of Pheo and Pheo loaded PEO-PS micelles, the CBF-leakage was of the same order of magnitude than the one of native LUVs, while this result increased in the case of Pheo loaded PEO-PCL micelles. As previously demonstrated, PEO-PCL micelles increased membrane permeability, we normalized the permeability after irradiation by the one before irradiation, in order to compare the effects of the two copolymer micelles on the membrane integrity (Figure 5b). The damages induced by PEO-PCL micelles on vesicles membranes are higher than those produced by PEO-PS micelles. 

Experiments using Anthracene-9,10-dipropionic acid (ADPA), present in solution, allowed us to define the singlet oxygen quantum yield of Pheo in solution and loaded in micelles (Figure 5c). As expected, the singlet oxygen quantum yield was higher in the case of Pheo loaded micelles with respect to that of free Pheo in solution where it forms inactive aggregates [28]. It has to be noted that the singlet oxygen production was the same independently on the nature of the polymer into the micelles.

Membrane permeability is influenced by the chemical species formed by oxidation through ^1^O_2_ produced by the irradiated photosensitizer [29]. In order to understand the chemical changes of DOPC due to oxidation, we analyzed the lipid composition after irradiation through mass spectrometry after separation with liquid chromatography (Figure 5d). The only two oxidized species found were DOPC with one or two peroxide chains at 818.6 Da (DOPC-(OOH)), and 850.6 Da (DOPC-(OOH)_2_), respectively (chemical structures in Figure S11). In order to gain quantitative kinetic insights into photo-induced lipid oxidation, we monitored the production of these two oxidized species as a function of the irradiation time (see Figure S12). Oxygen was gently bubbled through the system throughout the irradiation periods, and therefore we can assume the oxygen concentration to be constant. However, Pheo is subject to photo-bleaching, and we thus, have to account for its decreasing concentration over time. Pheo concentration ([Pheo]) was measured throughout the experiment by the absorbance of the solution at 670 nm where only Pheo absorbs, and the [Pheo] = f(t) was fitted by a second order polynomial. We then inferred the corresponding reaction kinetic constants using a simplified DOPC → DOPC-(OOH) → DOPC-(OOH)_2_ model as described below:

(9) $$Pheo\overset{\;\;\;\;\;}{\to }\;Photoproduct$$

(10) $$DOPC+Pheo\overset{\;\;k_{1}\;\;}{\to }\;DOPCOOH$$

(11) $$DOPCOOH+Pheo\overset{\;\;k_{2}\;\;}{\to }\;DOPC{\left(OOH\right)}_{2}.$$

This model was then analyzed using home-built software SA [30], which gave the rate constants for the first and second peroxidations. 

Experimentally, we found that only very low amounts of oxidized products were found after 30 min of irradiation. DOPC-(OOH)_2_ was still negligible after 30 min of irradiation, mainly DOPC-(OOH) was formed, but importantly the rate constant of the second oxidation reaction was higher than the rate constant of the DOPC-(OOH) formation (Figure 5d). We assumed that once oxidized, a lipid was more likely to receive a second peroxide, and therefore, led to a strong change in polarity.

Figure 3b indicated that LUVs exposed to empty PEO-PCL micelles, without light irradiation, underwent an alteration of their membrane integrity since leakage was observed over the time of incubation. To verify if empty PEO-PCL or PEO-PS micelles drastically altered cells, we analyzed cell viability after 24 h of incubation with these empty micelles in non-irradiated condition (Figure S13). No statistical difference was observed between PEO-PCL or PEO-PS micelles and control conditions. We concluded that both polymers did not affect cell viability.

### 2.4. Pheo Loaded PEO-PCL and PEO-PS Micelles Affect Significantly and Similarly Tumor Cell Viability after PDT Treatment in 2D and 3D Cell Cultures

**Figure 6:**
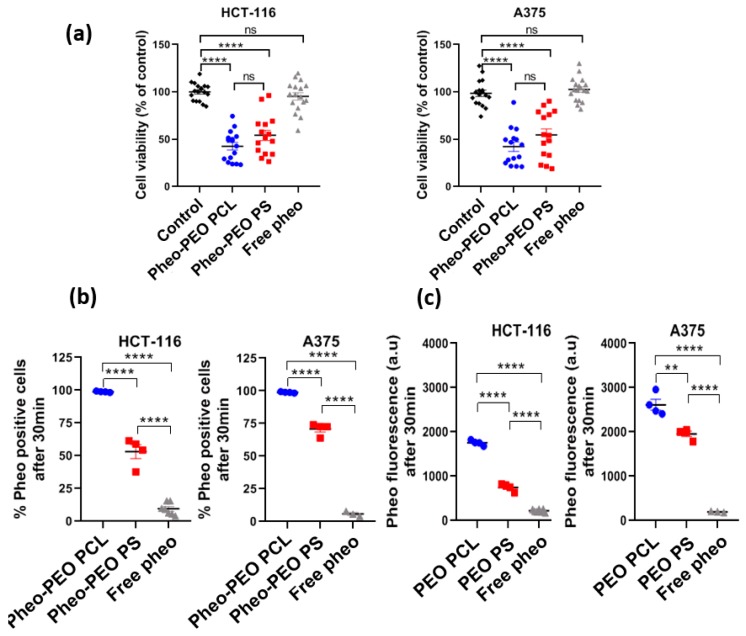
Assessment of tumor cell viability after PDT treatment with Pheo loaded PEO-PCL and PEO-PS micelles in 2D monolayers and flow cytometry analysis of Pheo levels in cells. (**a**) Cell viability was quantified 24 h after PDT treatment on monolayers using prestoblue assay. *n* = 3, *n* > 15. (**b** and **c**) After 30 min of incubation with Pheo loaded micelles or free Pheo, cells were analyzed by flow cytometry for positively labelled cells percentage (**b**) and the fluorescence intensity of Pheo in positively labelled cells (**c**). Statistical analysis by one-way ANOVA followed by Tukey’s multiple comparisons test. ns = non-significant; **** = *p* < 0.0001. Data are represented as the mean value ± SEM.

Viability of tumor cells, grown in 2D monolayers, was assessed 24 h after PDT treatment by a metabolic test named prestoblue assay, based on mitochondrial activity (Figure 6a). In functional experiments, cells were incubated with micelles for 30 min before being submitted to calibrated light irradiation. For this reason, a focus was made on flow cytometry experiments at 30 min of incubation (Figure 6b,c). In these conditions, Pheo was significantly more present in cells when carried by PEO-PCL micelles than by PEO-PS ones, both in terms of percentage of labelled cells and in terms of fluorescence intensity in the positively labelled cell population. Whichever micelles were used, encapsulation significantly improved Pheo delivery to cells compared to the free photosensitizer as less than 10% of cells were labelled in “free Pheo” condition.

Micelles of PEO-PCL and PEO-PS loaded with Pheo both induced a loss of cell viability of approximately 50% (Figure 6a). The difference between PEO-PCL and PEO-PS was not statistically significant, meaning that they induced cell death with similar efficiency after PDT treatment. The photosensitizer alone was incubated with the cells at the same concentration (0.33 µM) as the one used in the Pheo loaded micelles, but no cell death was measured. This result correlates with the weak percentage of cells labelled with Pheo measured by flow cytometry after 30 min of incubation (Figure 6b,c) and underlines the benefits of encapsulating the photosensitizer within micelles. The results obtained were similar for both colorectal HCT-116 and melanoma A375 cancer cell lines.

**Figure 7:**
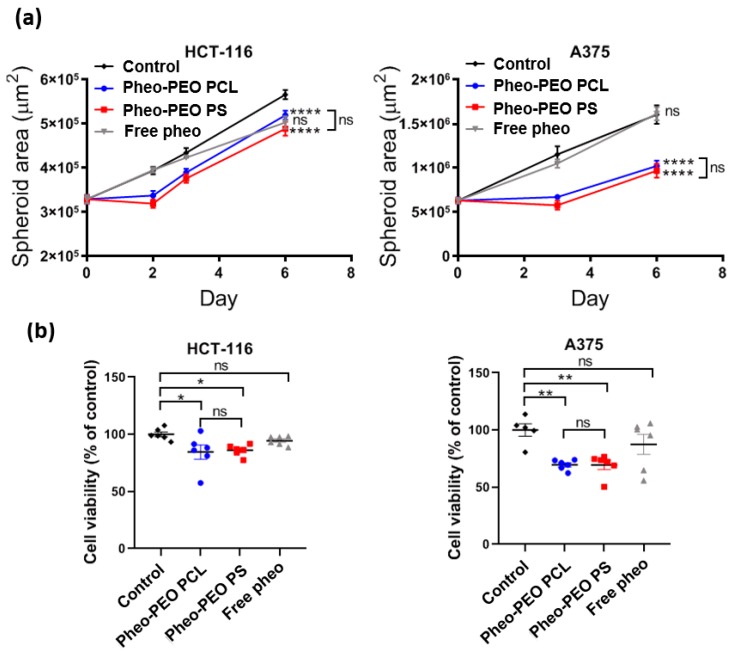
Assessment of tumor cell viability after PDT treatment with Pheo loaded PEO-PCL and PEO-PS micelles on 3D spheroids. (**a**) Growth curve of tumor spheroids after PDT treatment. *n* = 6. (**b)** Cell viability assessed 6 days after PDT treatment by intracellular ATP quantification on spheroids. *n* = 6. Statistical analysis by one (**b**) or two (**a**) –way ANOVA followed by Tukey’s multiple comparisons test. ns = non-significant; * = *p* < 0.1, ** = *p* < 0.05, **** = *p* < 0.0001. Data are represented as the mean value ± SEM.

**Figure A1:**
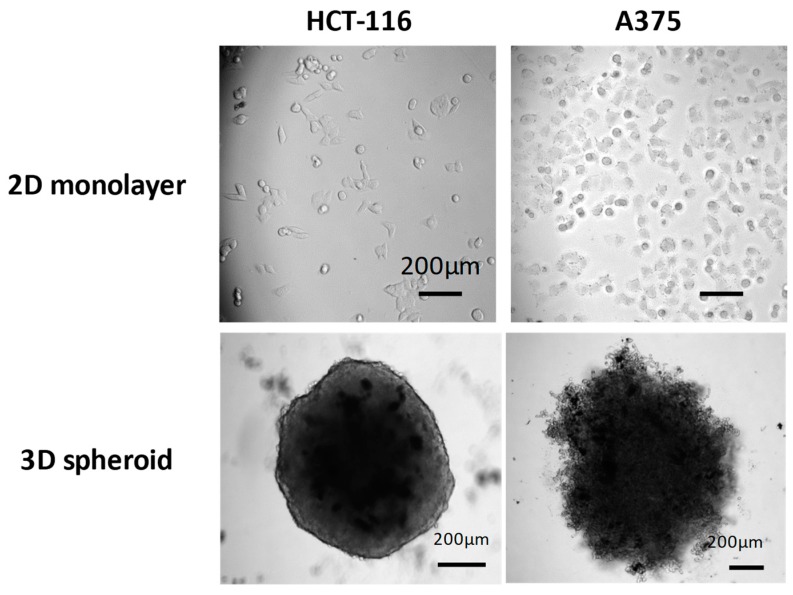
Morphological aspect of 2D monolayers and 3D tumor spheroids produced with human colorectal HCT-116 and human melanoma A375 cell lines.

We compared efficiency of Pheo loaded PEO-PCL or PEO-PS micelles in PDT of human 3D tumor spheroids (Figure 7). Both HCT-116 and A375 cell lines led to the production of 3D spheroids (Appendix A Figure A1), even if epithelial HCT-116 cells allowed the formation of rounder spheroids compared to the A375 ones. Their growth was followed over 6 days after a single PDT treatment with 3 µM Pheo loaded micelles or free Pheo (Figure 7a). While free Pheo did not affect the growth curve of spheroids, a growth delay was observed within two days after PDT treatment with Pheo loaded PEO-PCL or PEO-PS micelles. This growth delay was temporary, since spheroids kept growing after this latent phase. No statistical difference was observed between PEO-PCL and PEO-PS micelles. To confirm these optical observations, the intracellular ATP of the spheroids was measured 6 days after PDT treatment in each condition (Figure 7b), since ATP is generally considered a good indicator of cell viability [31]. No statistical difference was observed between the control and free Pheo conditions. Intracellular ATP was significantly lower in spheroids treated with Pheo loaded PEO-PCL and PEO-PS, even if no difference was observed between these two kinds of micelles.

## 3. Discussion

In this paper, studies of the cellular uptake of free Pheo show that its level of internalization in cells is low, and its photocytotoxicity is very limited (Figure 2, Figure 6 and Figure 7). In contrast, when Pheo is loaded in PEO-PCL or PEO-PS micelles, its cellular uptake is increased drastically, as well as its efficiency as a PDT sensitizer. This confirms the high benefit of copolymer micelles in Pheo delivery in cancerous cells for PDT applications as also mentioned in many previous studies [15,32]. 

If the advantages of drug encapsulation are well established, the mechanism by which amphiphilic block copolymer micelles deliver drugs is still poorly understood. In particular little is known experimentally about the interactions with plasma membranes and the cellular processes involved in the internalization of the drug. The initial encounter between nanovectors and membrane is crucial in determining the efficiency of the drug towards its cellular target. From a general point of view, nanovectors will not only lead to increased intracellular concentration of the drug, compared to unvectorized drugs, but could modify its subcellular localization and alter its natural targets. In this study, we focused on spherical PEO-PS and PEO-PCL micelles used to encapsulate Pheo, a lipophilic photosensitizer which generates reactive oxygen species by de-excitation from its excited triplet state. Pheo fluoresces at 670 nm is known to generate primarily singlet oxygen. This compound was used both as a model drug for photodynamic therapy applications, and also as a probe thanks to its natural fluorescence, which has given insight into the relationships between cellular uptake and therapeutic efficiency. The determination of the affinity constant of Pheo for both types of block copolymer micelles reveals that Pheo has a higher affinity for PEO-PS micelles. This may be partly explained by the strength of π-π interactions between Pheo and PS which can be stronger than the hydrophobic ones between Pheo and PCL [33]. This result suggests that Pheo will remain more easily sequestered in PEO-PS micelles than in PEO-PCL micelles. We then looked at the transfer efficiency of Pheo from micelles to DOPC LUVs, employed here as synthetic model membranes. The transfer of Pheo towards the LUVs was found to be independent of the type of micelle used, despite the differences in affinity constants. Interestingly, the presence of copolymer micelles has no effect on the thermodynamics of Pheo transfer to the LUV since the transfer constants, K_tr_, are comparable in all conditions.

Experiments on cellular uptake of Pheo loaded PEO-PCL and Pheo loaded PEO-PS (Figure 2) point out that whichever cancerous cell types were used, HCT-116 or A375, the Pheo incorporation is ~3 times higher after 4 h incubation when Pheo is encapsulated in PEO-PCL micelles than in PEO-PS micelles. It is important to note that with both cancerous cell types and both nanovectors, the cellular penetration of Pheo exhibits two phases, a fast one between 0 and 4 h of incubation and a slower phase leading to a plateau. This strongly suggests that the cellular penetration of encapsulated Pheo in PEO-PS or PEO-PCL involves the participation of endocytosis or another active mechanism.

100% of cells were positive for Pheo after 30 min of incubation when the carrier was PEO-PCL whereas this rate was only achieved at 4 h with PEO-PS, which indicates that Pheo loaded PEO-PCL may enter cells via different pathways compared to PEO-PS. 

It has to be noted that experiments on cells were conducted with an excess of photosensitizer with respect to the available bilayer volume. In this case, Pheo detected by flow cytometry is definitely not all located at the plasma membrane, but also intracellularly, as it was already proved by previous experiments on Pheo intracellular localization [18,34]. As FRET experiments showed that the Pheo absorbed on the membrane was the same in the presence of the two nanocarriers, we can reasonably conclude that PEO-PCL nanocarriers lead to higher Pheo cell internalization than PEO-PS ones.

Since both types of carriers share the same PEO corona, we can exclude specific attachment of the micelles to cells as a potential explanation for this marked difference. The hydrophobic core must thus play a crucial role in the incorporation mechanism. The PS core has been shown to be more robust towards destabilization than PCL one [18]. This could seem surprising since PCL is a semi-crystalline polymer and crystallinity has been shown to favor the stability of polymer micelles [35]. However, in the system presented here, we have previously demonstrated that whereas empty PEO-PCL retained some crystallinity, the introduction of Pheo induced its complete disappearance [34]. Furthermore, PS exhibits a much higher glass transition temperature (ca. 100 °C) compared to PCL (ca.−50 °C). At 37 °C, PCL is thus expected to be much softer than PS, providing higher mobility for all species. This has been very recently shown by Mosinger’s team who proved that oxygen was able to move more easily in a PCL matrix compared to a PS one [36]. This difference in characteristics of the micellar cores leads to differences of behavior when the micelles are in the presence of target membranes. Figure 3b describes the impact of block copolymer micelles on the permeability of LUVs, indicated by the time-dependence of the leakage of a small molecule. Leakage of carboxyfluorescein from LUVs was observed only in the case of PEO-PCL micelles which suggests defects in the lipid bilayer induced by PEO-PCL. Further, we observed a steady permeabilization rather than a short-lived destabilization. FRET experiments were ideal to tackle proximity problems such as this one. Figure 3a depicts the increase in rhodamine fluorescence due to FRET from NBD-labelled LUVs. The critical distance R_0_, at which FRET efficiency is reduced to 50% was calculated to be approximately 4 nm. Previous studies have shown [34] that the radius of the PEO-PCL micelle is ~ 9 nm according to neutron scattering measurements. These two parameters allow us to exclude the simple spatial proximity of unperturbed LUVs and micelles as a justification for the energy transfer. Rather, there has to be intermixing of copolymer into LUVs, or of labelled lipid into copolymer micelles. However, we cannot conclude on the true nature of this incorporation, and several models have been proposed [37]. The phenomenon observed in our case is much slower than in the study by Nawaz et al. [38], where they observed complete hemolysis in under 100 min using Pluronics as copolymers. The faster dynamics in the case of Pluronics are probably to be attributed to the higher critical micellar concentration of PEO-PPO-PEO than PEO-PCL, and the consequent higher concentration of free copolymer chains. These findings on model membranes have been confirmed by cellular studies on the uptake of rhodamine labelled PEO-PCL, and revealed the presence of polymer in the cells. For studies on both cell types, all cells were positive for rhodamine after very short interaction times thus supporting the hypothesis of polymer incorporation into cells. However, the intensity of the rhodamine fluorescence, which is representative of polymer incorporation, is steadily increasing even after 24 h of interaction. This indicates the contribution of a passive mechanism, given that saturation is not reached after such a long interaction time.

In order to study the effects of singlet oxygen production in the vicinity of lipid bilayers, we performed LUV leakage experiments under irradiation. We observed that light irradiation of Pheo loaded micelles led to an increase of LUV leakage rate, more so in the case of PEO-PCL micelles than PEO-PS micelles and free Pheo. This superior effect for PEO-PCL micelles is observed in spite of similar singlet oxygen production for both types of micelles, which is another indication of the active part played by the PEO-PCL copolymer in destabilizing the lipid membrane. Further, we showed that the rate of photo-induced peroxidation was consistently higher when Pheo was carried by PEO-PCL micelles, again confirming the active role played by the polymer. Significant peroxidation was only observed after prolonged irradiation (several hours, Figure S12). Simple lipid peroxidations cannot lead to LUV permeabilization [39]. Recently Bachelar et al. [40] proposed that direct contact between photosensitizers and lipids were essential for the progress of lipid oxidation and consequently for the formation of aldehydes leading to truncated lipids and membrane permeabilization. We did not detect truncated lipids, even though Pheo encapsulated in PEO-PCL may be in direct contact with DOPC upon the intermixing of copolymer with LUVs. 

Very interestingly, these studies on model membrane systems, which help us to confirm the active part played by the co-polymer carrier, are counterbalanced by cellular studies in PDT conditions. By flow cytometry, we showed that incubating cells for 30 min with Pheo loaded PEO-PCL resulted in a much greater concentration of photosensitizer within, or attached to the cells, compared to Pheo loaded PEO-PS micelles. One could expect that delivery by PEO-PCL micelles would result in greater photocytotoxic activity of Pheo. Surprisingly, on 2D cell cultures of both cancerous cell lines tested, we find similar phototoxic efficiencies irrespective of the type of nanovector, with around 50% cell viability retained after light activation of the photosensitizer. 

Several hypotheses can be formulated to explain these results. Pheo delivered by PEO-PCL could enter cells via more than one pathway: mass transfer between micelles and cell where both polymer and cargo can be incorporated into the membrane (as shown in Figure 3); direct transfer of Pheo from micelle to cell membrane, as proposed by Kerdous et al. [27] and Till et al. [34]; by endocytosis [34]. In the case of Pheo delivered by PEO-PS micelles, direct jump and endocytotic mechanisms seem to be the main cell entrance pathways. Very recently Kubát et al. [36] clearly showed that the lifetime of singlet oxygen produced by photosensitizer encapsulated in PS is higher (6.4 µs to 16 µs) than that photo-generated in PEO-PCL micelles (3.6–3.8 µs). In PEO-PS, singlet oxygen due to its longer lifetime will have time to diffuse through the nanovector in the vicinity of numerous intracellular targets and to photo-oxidize them. In PEO-PCL, the shorter singlet oxygen lifetime leads to a decrease of the amount of cellular photodamage produced, compared to those by Pheo loaded PEO-PS system. Altogether, these experiments tend to show that the microenvironment of Pheo will govern its photo-toxicity via the rate of singlet oxygen deactivation. The superior apparent efficiency of Pheo loaded PEO-PS micelles indicates that a significant part enters cells unperturbed. Finally the increased uptake of PEO-PCL might be compensated by the higher activity of the Pheo in the PEO-PS.

On 3D spheroid models, which are considered more relevant to in vivo situations, comparable phototoxicities were observed with both loaded PEO-PCL and PEO-PS micelles. The observed decrease in spheroid volume could be attributed to the death of the peripheral cell layer as previously described [41]. At 2 days post irradiation, growth of the spheroids was restored. At 6 days post irradiation, spheroid cells had largely recovered their viability. This interesting result suggests that several irradiations might be necessary for a total eradication of the cancerous mass. Three-dimensional tumor spheroids, which simulate avascular tumor regions were found to be highly useful for understanding PDT mechanisms [42]. In particular, studies on tumor spheroids have been helpful in visualizing and modeling uptake and penetration of photosensitizers through multiple cell layers. The majority of studies on free or encapsulated photosensitizer indicated nearly exclusive peripheral uptake, within the first layers of the spheroid [21,43,44]. However, some photosensitizers such as EtNBS seemed to demonstrate a higher affinity for the acidic core of spheroids due to its cationic nature, which enables its efficient diffusion toward low pH gradients [45,46]. Since the vast majority of photosensitizers do not present this affinity for the acidic core of the spheroids, and localize preferentially on the outermost cell layers, multiples PDT treatments are useful for targeting more cell layers until the total eradication of tumor mass.

## 4. Materials and Methods 

### 4.1. Materials

Poly (ethylene oxide)-b-poly (caprolactone) (PEO-PCL) 5k-4k, poly (ethylene oxide)-b-poly (styrene) (PEO-PS) 2,3k-3,1k were purchased from Polymer Source (Polymer Source, Inc., Dorval, Canada). 1,2-Dioleoyl-sn-glycero-3-phosphocholine (DOPC) and 1,2-dioleoyl-sn-glycero-3-phosphoethanolamine-N- (lissamine rhodamine B sulphonyl) (ammonium salt) (Lys- Rhod-PE) come from Avanti Polar Lipids (Avanti Polar Lipids, Inc., USA). (N-(7-Nitrobenz-2-Oxa-1,3-Diazol-4-yl)-1,2-Dihexadecanoyl-sn-Glycero-3-Phosphoethanolamine, Triethylammonium Salt) was bought from Thermo Fisher (NBD-PE, N360, Waltham, MA, US). Pheophorbide-*a* was purchased from Wako (Japan). Chloroform, methanol, acetone, phosphate buffer (PBS, P4417) and carboxyfluorescein (CBF, 21877) were purchased from Sigma Aldrich (Merck, Darmstadt, Germany). The water used to prepare all PBS solutions was ultrapure water at 18 MΩ resistivity obtained with an ELGA Purelab Flex system (ELGA LabWater, High Wycombe, UK). Anthracene-9,10-dipropionic acid disodium salt (ADPA) was purchased from Santa Cruz Biotechnology (Dallas, TX, US).

### 4.2. Preparation of block-copolymer micelles and Large Unilamellar Vesicles

Block-copolymer micelles were prepared as reported elsewhere [18]. A total of 20 mg of the copolymer were dispersed in 400 μL of acetone and this solution is slowly added to 5 mL of water whilst stirring. Acetone is left to evaporate during 48 h. For all samples loaded with Pheo, a solution of the photosensitizer in acetone is used to dissolve the polymer prior to its addition on the water solution. The molar ratio between the photosensitizer and the polymer was kept constant at 1/30 mol/mol to ensure the complete encapsulation. The hydrodynamic sizes of the carriers were determined by Dynamic Light Scattering (DLS) on a Malvern Zetasizer instrument, using the General Purpose, non-negative least square routine for data processing. PEO-PCL micelles had intensity-mean hydrodynamic diameter d_h_ = 29 ± 9 nm and PEO-PS micelles had d_h_ = 21 ± 7 nm.

The same protocol was used in the case of rhodamine labelled PEO-PCL. In that case, a 5mg/mL acetone stock solution of the rhodamine labelled copolymer (PEO-PCLrhod) was prepared and 400 μL of this solution were added to 20 mg of PEO-PCL to give 10% of labelled polymer in the micelles.

The Large unilamellar vesicles (LUVs) also called liposomes were prepared according to the traditional method of hydration of a dry lipid film followed by extrusion. First, the lipid is solubilized in chloroform in a haemolysis tube (20 mg·mL^−1^). 1 mL of chloroform is evaporated first with a rotary evaporator followed by dynamic vacuum for a minimum of 4 h. Once the film is dry, 2 mL of filtered PBS is added to the tube to obtain a suspension of lipids at a concentration of 10 mg·mL^−1^. For leakage experiments described in Section 4.6 the dry film is hydrated with 2 mL of 60 mM CBF in PBS. After vortexing if necessary to finish peeling off the film from the bottom of the tube, the lipid suspension was put in a bath at 60 °C for 30 min and then for one h in an ultrasonic bath at 60 °C. The lipid suspension is then extruded through a 100 nm pore membrane (Nucleopore Track-Etch Membrane, Whatman, UK) using a hand extruder (Avanti Mini Extruder, Avanti Polar Lipids, Alabaster, AL, USA) to obtain a uniform LUV distribution. In the case of LUVs prepared for leakage experiments, steric exclusion separation was used and the samples were eluted twice through a column filled with Sephadex G 50 (GE Healthcare—Life Sciences, Pittsburgh, PA, USA) in order to separate the CBF loaded LUVs from the free CBF in solution. All fractions were controlled in DLS and all those containing LUVs were pooled. The consequent dilution of the samples was calculated by making the ratio of the total volume recovered and the volume of suspension initially injected into the column.

LUVs size was verified by DLS using the same routine as described above, their hydrodynamic size typically being around 100 nm in diameter. DOPC LUVs were used for the affinity constant measurements. In the case of FRET measurements DOPC LUVs contained 1% mol/mol of Liss Rhod PE or NBD-PE.

### 4.3. Association Constants

Association constants were derived by measuring the fluorescence emission of Pheo in the presence of different concentrations of the copolymer micelles. 

For each series of measurements, a Pheo stock solution at 2 × 10^−8^ M in PBS was prepared by adding 30 μL of a solution of Pheo in acetone ([Pheo] = 0.01 mg·mL^−1^) to 25 mL of PBS. After stirring, the solution was flushed with argon for 10 min to drive off residual acetone and dissolved oxygen. Samples were prepared by adding different amounts of copolymer micelles (volumes between 5 and 500 μL of the stock copolymer solution, [copolymer] = 4 mg·mL^−1^), to 1 mL of the Pheo stock solution. The final total volume of 2 mL was reached by completing with PBS. Polymer concentrations varied between 1 and 100 µM and Pheo concentrations between 0.033 and 33 µM. All samples were bubbled with argon for 1–2 min to remove dissolved oxygen and then closed and incubated in the dark for a minimum of 3 h. After incubation, the fluorescence of the samples was measured by exciting them at 400 nm and measuring their emission from 600 to 780 nm. The fluorescence intensity at 675 nm was plotted as a function of the copolymers concentration in the samples. Measurements were repeated at least twice for each sample.

### 4.4. Förster Resonance Energy Transfer Measurements (FRET)

#### 4.4.1. Pheo/rhodamine FRET

The samples containing a fixed rhodamine labelled liposomes concentration (0.01 mg·mL^−1^) and variable amounts of copolymer micelles loaded with Pheo (from 10 to 75 μL of a [copolymer] = 0.08 mg·mL^−1^ and from 15 to 50 μL of a [copolymer] =0.8 mg·mL^−1^) were used in order to change the molar ratio between copolymer and DOPC lipid from 0.03 to 1.8. In these samples, the rhodamine concentration was 0.12 μM (i.e., ~1% mol of lipid content).The samples were prepared by mixing the stock solutions in a 96-well plate (total volume 200 μL) and the fluorescence of the samples was followed for 10 h by exciting them at 560 nm and measuring their emission at 592 nm on a Synergy H1 plate reader (Biotek). For each studied system, samples at the same concentrations without LUV were measured and their fluorescence was subtracted from that of the micelles/LUV mixtures. The fluorescence values at 6 h were used for the analysis described above.

#### 4.4.2. NBD/rhodamine FRET

Samples containing a fixed NBD-labelled LUVs concentration (0.0025 mg·mL^−1^) and variable amounts of micelles (from 10 to 250 μL [polymer] = 4 mg·mL^−1^) were prepared in order to change the molar ratio between copolymer and DOPC lipid from 0.6 to 16 and allowed to incubate for about 4 h. In these samples, the NBD concentration was 0.032 μM (i.e., ~1% mol of lipid content). After incubation, the fluorescence of the samples was measured by exciting them at 464 nm and measuring their emission from 470 to 750 nm. On these emission spectra, two peaks of interest appear: one at 578 nm belonging to the rhodamine labelled copolymer which partially overlaps to the 540 nm one relative to NBD in LUVs. For each studied system, samples at the same concentrations without liposomes were measured and their fluorescence was subtracted from that of the micelles/LUV mixtures. 

We analysed the data as for the Pheo/Rhodamine FRET experiments and similarly to equations [5] and [6], we can write the equilibrium,

(12) PEOPCLrhod+NBD⇆PEOPCLrhod:NBD

where *PEO-PCLrhod* and NBD are the rhodamine and NBD at distances larger than 4 nm and *PEO-PCLrhod:NBD* is the rhodamine/NBD couple undergoing to resonance energy transfer. When NBD is excited at 464 nm, the rhodamine signal appears only when the FRET occurs. In a similar way as before, the associated constant is written as,

(13) $$k_{tr}=\frac{\left[PEOPCLrhod:NBD\right]}{\left[PEOPCLrhod\right]\times \left[NBD\right]}$$

and obtained from the plot of the rhodamine maximum fluorescence intensity (λ = 578 nm) increase as a function of copolymer concentration (see Figure S10). As in [7] we can express the fluorescence signal as a function of the concentration of each species and a fluorescence proportionality constant:

(14) $$F_{\lambda }=F_{NBD,\lambda }+F_{PEOPCLrhod:NBD,\lambda }=f_{NBD,\lambda }\left[NBD\right]+f_{PEOPCLrhod:NBD,\lambda }\left[PEOPCLrhod:NBD\right].$$

$F_{NBD,\lambda }$, $F_{PEOPCLrhod:NBD,\lambda }$ are the fluorescence intensity of NBD and PEO-PCLrhod coupled to NBD at a given wavelength. $f_{NBD,\lambda }$ and $f_{PEOPCLrhod:NBD,\lambda }$ are the fluorescence proportionality constants linking NBD and PEO-PCLrhod:NBD to their concentration. $f_{NBD,\lambda }$ is experimentally evaluated from the values of the spectrum in the absence of the fluorescent polymer. $f_{PEOPCLrhod:NBD,\lambda }$ and $k_{tr}$ are the fitted parameters.

### 4.5. Singlet Oxygen Quantum Yield

The singlet oxygen quantum yield has been measured for each system (pheo in PBS, Pheo loaded PEO-PS and Pheo loaded PEO-PCL) though an indirect method by using a water-soluble anthracene, ADPA. In order to get the singlet oxygen quantum yield ($\Phi_{\Delta }$) methylene blue was used as a reference photosensitizer with a known $\Phi_{\Delta }^{MB}=0.52$ in water. [Pheo] = [methylene blue] = 1 μM, ADPA concentration was 20 μM. In the presence of singlet oxygen, ADPA is oxidized to form an endoperoxide which no longer absorbs between 300 and 420 nm. We then monitored the UV absorbance at 400 nm during irradiation with a LED at 656 nm (35 mW), the decrease of ADPA absorbance as a function of time was followed during 1 h and was modelled with an exponential function to determine its characteristic oxidation rate. 

The quantum yields of singlet oxygen production, $\Phi_{\Delta }^{pheo}$, are calculated in the case of Pheo in PBS, and Pheo loaded in the copolymer micelles according to:

(15) $$\Phi_{\Delta }^{pheo}=\Phi_{\Delta }^{MB}\left(\frac{m_{pheo}}{m_{MB}}\frac{F_{MB}}{F_{pheo}}\right).$$

$m_{pheo}$ and $m_{MB}$ represent the photobleaching rates of Pheo and methylene blue, respectively; $F_{pheo}$ and $F_{MB}$ are the optical densities at irradiation wavelength of Pheo and methylene blue, respectively ($F=1-10^{-Abs\left(656\right)}$).

All UV-Visible absorbance measurements (also those during irradiation) were performed using a diode array spectrometer (HP 8452A, Hewlett-Packard, Hayward, CA, USA) driven using SpectralWork software (Olis, Bogart, GA, USA).

### 4.6. Leakage Experiments

LUVs loaded with CBF were prepared as described above. LUVs containing CBF were mixed with Pheo loaded or not in the copolymer micelles (Pheo loaded PEO-PCL, Pheo loaded PEO-PS, and free Pheo). The total volume of the resulting PBS solution was 2 mL and [DOPC] = 120 μM, [Pheo] = 1 μM. After mixing, the solutions were incubated for 30 min in the dark. After incubation, the mixture was stirred and irradiated for 30 min using an LED at 656 nm (35 mJ·cm^−^²). The fluorescence of the samples was measured by exciting the CBF at 492 nm and looking at the emission between 500 and 800 nm. The fluorescence was measured before and after the addition of micelles or Pheo solution, and then for 15–20 h after irradiation. After this time, 100 μL of 10% Triton × 100 in PBS were added to the mixture to destabilize the liposomes and release all the encapsulated CBF. Fluorescence was measured again. 

The leakage as a function of time is defined as the CBF fraction which exits the liposomes:

(16) $$Leakage\left(t\right)=\frac{I\left(t\right)-I_{0}}{I_{Triton}-I_{0}}.$$

I(t) is the fluorescence intensity (λ = 518 nm) of the mixture at a given time and is proportional to the CBF concentration outside the LUVs. I(t) is normalized by the fluorescence intensity (λ = 518 nm) after addition of Triton X, $I_{Triton}$, which is proportional to the total amount of CBF initially inside the LUVs. 

Both $I_{Triton}$ and I(t) are corrected by $I_{0},$ the fluorescence intensity (λ = 518 nm) before adding the micelles or the Pheo solution so to take into account the possible presence of residual CBF outside the liposomes. 

The follow-up of the leakage as a function of time allows us to calculate the permeability coefficient, P, of the liposomes using the formula,

(17) $$\mathrm{Leakage}\left(t\right)={\mathrm{Leakage}}_{\max}\left(1-e^{\frac{t}{\tau_{\mathrm{leak}}}}\right)$$

with

(18) $$P=\frac{r}{3\tau_{leak}}$$

where $Leakage_{max}$ is the system’s maximum leakage at t ∞, $\tau_{leak}$ is the characteristic time of leakage, and r the liposomes radius.

### 4.7. Oxidation Experiments

For lipid oxidation experiments, DOPC LUVs and co-polymer micelles were prepared as described above. For typical experiments, LUVs were at a concentration such that [DOPC] = 300 µM, and micelles at a concentration such that [Pheo] = 1 µM, in a total volume of 2 mL of PBS. One sample was prepared for each time of irradiation studied. Oxygen was gently bubbled in the samples throughout irradiation so as to maintain its concentration constant. The red LED, used to irradiate, was set at 35 mJ·cm^−^². Following irradiation, the samples were bubbled with Argon to replace O_2_ and stored at 4 °C in the dark. The lipids were extracted from this medium following Bilgh and Dyer. 1 mL of MeOH was added to each sample, followed by 2 mL of CHCl_3_ and vigorous shaking. The organic phase was collected and dried. The solid residue was then dissolved in 1 mL of MeOH and diluted until a theoretical concentration of [lipid] = 0.24 mM was obtained. A total of 3 µL of these solutions were then injected in an Acquity UPLC-MS chain (Waters, Manchester, UK) equipped with a BEH-C8 column (1.7 mm; 2.1 × 50 mm). Elution was achieved with a gradient of 8 mM ammonium acetate and methanol. Total run time was 19 min. Detection and quantification was achieved using a Xevo G2 QTOF mass spectrometer (Waters, UK). The QTof-MS was operated in the positive-ion mode with a capillary voltage of 2.5 kV, a cone voltage of 50 V, the cone gas flow of 20 L·h^−1^, the source temperature set to 130 °C and the desolvation temperature set to 450 °C.

### 4.8. Cell Culture

Human HCT-116 colorectal cancer cells (CCL-247) and A375 melanoma cancer cells (CRL-1619) were purchased from ATCC. Cells were grown in DMEM containing 4.5 g·L^−1^ glucose, GLUTAMax and pyruvate, supplemented with 10% of heat-inactivated fetal bovine serum and 100 U/mL penicillin, and 100 µg·mL^−1^ streptomycin. Cells were maintained at 37 °C in a humidified atmosphere containing 5% CO_2_. Cell culture media were changed three times a week. Both cell types tested negative for mycoplasma using MycoAlert mycoplasma detection kit (Lonza, Basel, Switzerland) all along the experiments.

### 4.9. Determination by Flow Cytometry of Photosensitizer Penetration in Cells Grown in Monolayer 

The day prior to experimentation, 80,000 cells were seeded in 12-well plates. These cells were incubated for 24 h, 6 h, 4 h, 2 h, 1 h and 30 min with the two different types of Pheo loaded micelles (PEO-PCL, PEO-PS) or with free Pheo. 4 biological replicates were used in each condition. In this experiment, the final concentration of Pheo was set at 0.33 µM, which equals 10 µM of polymer. After the last incubation time, cells were washed with 2 mL of PBS, gently trypsinized and dispatched in a 96-well plate U bottom, kept on ice bed. Cells in suspension were then analyzed on a BD LSRFortessa X-20 flow cytometer, equipped with an HTS module for plate reading. At least 20,000 events were acquired in each well. Flow cytometry allows the quantification of Pheo fluorescence within the cells, meaning when localized either in the plasma membrane or intracellularly. Using this method, no speculation can be made on whether Pheo is still associated with the polymers or free in the cell.

### 4.10. Determination by Flow Cytometry of Rhodamine-PEO PCL Polymer Penetration in Cells Grown in Monolayer 

As described in Section 4.9, 80,000 HCT-116 or A375 cells were seeded in 12-well plates the day before the experiment. The cells were incubated for 24 h, 6 h, 4 h, 2 h, 1 h and 30 min with micelles produced with PEO-PCL, covalently-linked to rhodamine, at a ratio of 1/10 with unlabeled PEO-PCL. 4 biological replicates were used in each condition. In this experiment, the final concentration of PEO-PCL polymer was set at 10 µM, as in the previous experiment on photosensitizer penetration quantified by flow cytometry. After the last incubation time, cells were washed with 2 mL of PBS, gently trypsinized and dispatched in a 96-well plate U bottom, kept on a bed of ice. Cells in suspension were then analyzed on a BD LSRFortessa X-20 flow cytometer, equipped with an HTS module for plate reading. At least 20,000 events were acquired in each well. Flow cytometry allows the quantification of Rhodamine-PEO-PCL polymer fluorescence within the cells (either in the plasma membrane or intracellular). Using this method, no speculation can be made on whether PEO-PCL is still self-assembled as micelles or dispersed in unimer form.

### 4.11. Cell Viability Assessement after Photodynamic Therapy of Monolayers

The day before the experiments, 4,000 HCT-116 or A375 cells were seeded in 96-well plates. The day of experiment, cells were incubated with empty PEO-PCL micelles, Pheo loaded PEO-PCL micelles, empty PEO-PS micelles, Pheo loaded PEO-PS micelles or free Pheo for 30 min at 37 °C. Concentration of Pheo was set to 0.33 µM and polymer concentration was accordingly set to 10 µM. In light-irradiated conditions, after this 30 min incubation time with micelles, the 96-well plate was deposited on an overhead projector lamp with a band-pass filter (λ > 400 nm). Cells were photo-irradiated for a total of 4 min (2 min light on, 2 min light off and then 2 min light on). Each well received 8.2 J·cm^−2^. After illumination, cells were incubated for 24 h at 37 °C. Viability was then assessed using PrestoBlue reagent (Invitrogen, Carlsbad, CA, USA) according to the manufacturer’s instructions, and read on plate reader Synergy H1 (Biotek, Winooski, VT, USA).

### 4.12. Photodynamic Therapy of 3D Tumor Spheroids

HCT-116 and A375 spheroids were incubated with Pheo loaded PEO-PCL micelles, Pheo loaded PEO-PS micelles or free Pheo for 30 min at 37 °C. Concentration of Pheo was set at 3 µM as previously described for PDT in 3D spheroids [21]. Spheroids were irradiated for 8 min (4 min light on, 4 min light off and then 4 min light on) using the same overhead projector lamp as for irradiation of monolayers. Spheroids treated by PDT were observed by optical microscopy (AxioObserver A1 equipped with a 5× plane N objective in phase contrast) and photographed over one week and their areas were determined using Image J software (NIH, Bethesda, MD, USA) as previously described [18,47].

### 4.13. Cell Viability Assessement after Photodynamic Therapy of Spheroids

The growth curve of spheroids was followed macroscopically over six days. Six days after PDT treatment, the cell viability of spheroids was assessed by quantifying intracellular ATP content using CellTiterGlo3D luminescent kit (Promega, Madison, WI, USA), according to the manufacturer protocols, as previously described [48]. Briefly, after taking the last picture of the growth curve, cell culture medium was removed so that only 50 µL of supernatant were left with the spheroid in the well. The same volume of reagent was added and incubated under gentle shaking at room temperature for 30 min. After spheroid lysis, the whole content of each well (i.e., 100 µL) was transferred into a white-96-well plates and luminescence was read on plate reader Synergy H1 (Biotek).

### 4.14. Statistics

The number of biological replicates (*n*) and number of independent repetitions of the experiments (N) is indicated in the figure legends. Statistical differences between values were assessed either by one-way or two-way ANOVA, followed by Tukey’s multiple comparison test, which compares the mean of each condition with all other conditions. The statistical analysis were performed with GraphPad Prism 8 (GraphPad Software, San Diego, CA, USA).

## 5. Conclusions

We showed that Pheo loaded PEO-PCL micelles allow for a greater cellular uptake of photosensitizer than Pheo loaded PEO-PS micelles. However, this higher apparent concentration does not lead to lower cell viability, which confirms that chemical environment, as well as photosensitizer concentration, are significant parameters for phototoxicity. The combination of physico-chemical and biological experiments suggests that PEO-PCL micelles could be incorporated into membranes, which leads to a decrease in efficiency, while in contrast, PEO-PS micelles do not incorporate into membranes. This leads to optimized photosensitizer activity. Studies on both 2D and 3D cell cultures confirm that encapsulation of photosensitizer is recommended and repeated irradiations are necessary to improve PDT efficiency.

## Supplementary Materials

The following are available online at https://www.mdpi.com/2072-6694/12/2/384/s1, Figure S1: Representative fluorescence spectra and extrapolated intensity at 675 nm, Figure S2: Absorbance spectrum of Pheophorbide a in various environments, Figures S3 to S6: Fluorescence intensity decays of Pheophorbide a in ethanol, in PEO-PCL micelles, in PEO-PS micelles, in PBS, Figure S7: Fluorescence and absorbance spectra of Pheophorbide a and Rhodamine, Figure S8: Fluorescence intensities of DOPC-Rho LUVs and Pheo loaded micelles mixtures, Figure S9: Size Exclusion chromatography, Figure S10: Fluorescence spectra for mixtures of NBD-labelled liposomes and Rhodamine-labelled micelles, Figure S11, Chemical structures of oxidised DOPC, Figure S12: integrated peak areas from mass spectrometry of oxidised lipids, Figure S13: HCT-116 tumor cell viability, Table S1: Life time values of Pheophorbide fluorescence, Table S2: Calculation of R0 values obtained for the FRET couples Rhodamine b/Pheo and NBD/Rhodamine b.
